# POSEIDON: Peptidic Objects SEquence-based Interaction with cellular DOmaiNs: a new database and predictor

**DOI:** 10.1186/s13321-024-00810-7

**Published:** 2024-02-16

**Authors:** António J. Preto, Ana B. Caniceiro, Francisco Duarte, Hugo Fernandes, Lino Ferreira, Joana Mourão, Irina S. Moreira

**Affiliations:** 1 Center for Neuroscience and Cell Biology, University of Coimbra, 3004-504 Coimbra, Portugal; 2 PhD Programme in Experimental Biology and Biomedicine, Institute for Interdisciplinary Research (IIIUC), University of Coimbra, Casa Costa Alemão, 3030-789 Coimbra, Portugal; 3 Department of Life Sciences, University of Coimbra, Calçada Martim de Freitas, 3000-456 Coimbra, Portugal; 4 CNC - Center for Neuroscience and Cell Biology, CIBB - Centre for Innovative Biomedicine and Biotechnology, University of Coimbra, Coimbra, Portugal; 5 FMUC - Faculty of Medicine, University of Coimbra, Coimbra, Portugal; 6 MIA – Multidisciplinary Institute of Ageing, University of Coimbra, Coimbra, Portugal

**Keywords:** Cell-penetrating peptide, Database, Cargo delivery, Quantitative uptake, Uptake efficiency

## Abstract

Cell-penetrating peptides (CPPs) are short chains of amino acids that have shown remarkable potential to cross the cell membrane and deliver coupled therapeutic cargoes into cells. Designing and testing different CPPs to target specific cells or tissues is crucial to ensure high delivery efficiency and reduced toxicity. However, in vivo*/*in vitro testing of various CPPs can be both time-consuming and costly, which has led to interest in computational methodologies, such as Machine Learning (ML) approaches, as faster and cheaper methods for CPP design and uptake prediction. However, most ML models developed to date focus on classification rather than regression techniques, because of the lack of informative quantitative uptake values. To address these challenges, we developed POSEIDON, an open-access and up-to-date curated database that provides experimental quantitative uptake values for over 2,300 entries and physicochemical properties of 1,315 peptides. POSEIDON also offers physicochemical properties, such as cell line, cargo, and sequence, among others. By leveraging this database along with cell line genomic features, we processed a dataset of over 1,200 entries to develop an ML regression CPP uptake predictor. Our results demonstrated that POSEIDON accurately predicted peptide cell line uptake, achieving a Pearson correlation of 0.87, Spearman correlation of 0.88, and r^2^ score of 0.76, on an independent test set. With its comprehensive and novel dataset, along with its potent predictive capabilities, the POSEIDON database and its associated ML predictor signify a significant leap forward in CPP research and development. The POSEIDON database and ML Predictor are available for free and with a user-friendly interface at https://moreiralab.com/resources/poseidon/, making them valuable resources for advancing research on CPP-related topics. Scientific Contribution Statement: Our research addresses the critical need for more efficient and cost-effective methodologies in Cell-Penetrating Peptide (CPP) research. We introduced POSEIDON, a comprehensive and freely accessible database that delivers quantitative uptake values for over 2,300 entries, along with detailed physicochemical profiles for 1,315 peptides. Recognizing the limitations of current Machine Learning (ML) models for CPP design, our work leveraged the rich dataset provided by POSEIDON to develop a highly accurate ML regression model for predicting CPP uptake.

**Graphical Abstract:**

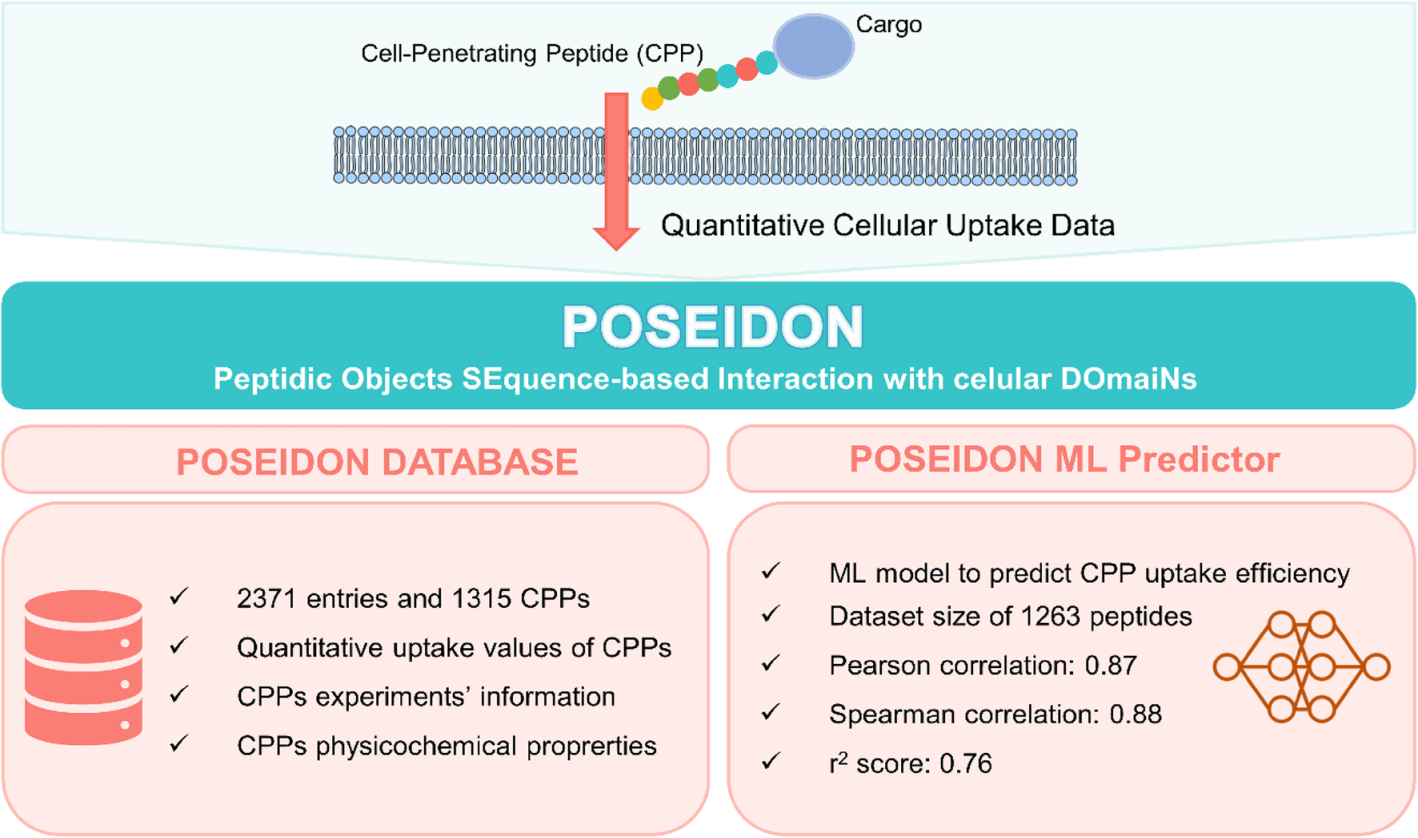

**Supplementary Information:**

The online version contains supplementary material available at 10.1186/s13321-024-00810-7.

## Introduction

The biomedical field faces a significant challenge in the development of pharmacological compounds that can be efficiently delivered to binding sites. Cell-Penetrating Peptides (CPPs) provide a safe and effective means of delivering therapeutic agents and other cargoes into cells without causing damage to the cell membrane. Such cargo may include nucleic acids, proteins, peptides, nanoparticles, fluorophores, small therapeutic compounds, and peptide nucleic acids [1–4]. CPPs share common structural and physicochemical features, including short amino acid sequences consisting of 4–40 residues, which typically adopt α-helical structures [1, 5–7]. They are often amphiphilic or cationic, soluble in water, partially hydrophobic, and rich in arginine and lysine residues [6, 8–10].

CPPs have been extensively studied for their potential use as drug delivery systems and diagnostic tools in various medical areas, such as immunotherapy [11], neurological disorders [12], and cancer [13]. Although the number of clinical trials involving CPPs has increased, only one CPP has been approved by the European Medicines Agency (EMA) [1, 14]. The design and testing of different CPPs in vitro and in vivo can be expensive and labor-intensive [15, 16]. Therefore, efficient computational tools and methodologies are necessary for rapid and accurate identification of suitable CPPs. Recently, many computational resources have been used to provide information on CPPs design and uptake ability, including Machine Learning (ML) approaches such as C2Pred [17], CPPred-RF [18], SkipCPP-Pred [19], CellPPD-MOD [20], ML-based prediction of CPP (MLCPP) [21, 22], Kernel Extreme Learning Machine-based prediction (KELM-CPPpred) [23], and StackCPPred [24]. However, existing methods rely solely on classification approaches because of the limited qualitative nature of the data available in current databases. One of the most commonly used databases, CPPsite 2.0, published in 2016, contains qualitative data for over 1,800 CPPs sequences [2].

We created POSEIDON–Peptidic Objects SEquence-based Interaction with cellular DOmaiNs, a comprehensive database containing quantitative uptake values and physicochemical properties of 1,315 cell-penetrating peptides across various scenarios, to fill gaps in the current CPP design. POSEIDON is indeed the most extensive database of quantitative CPP uptake values, with up-to-date information and unique data collection. Furthermore, POSEIDON includes a processed dataset that employs a well-designed methodological approach, making it an ideal benchmark for the development of new ML algorithms. By leveraging this database, coupled with cell line genomic features, we developed a novel ML regression model that accurately predicted CPP uptake efficiency.

## Methods

### Data extraction and curation

**Fig. 1:**
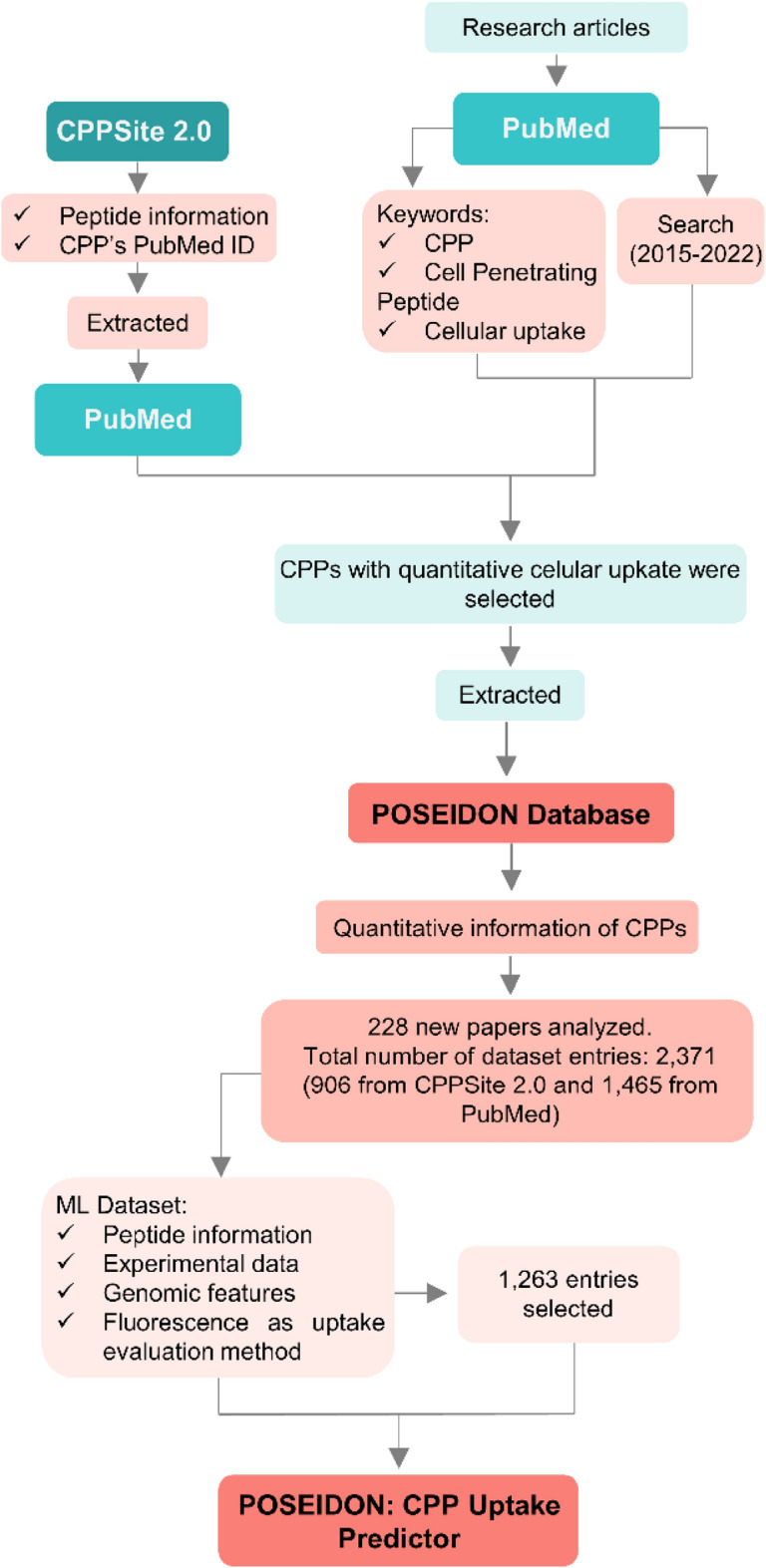
Overall workflow of data collection

The general workflow for data collection is shown in Fig. 1, which depicts the collection, organization, and extraction of accurate and relevant information from various sources to create a centralized and annotated database. CPP sequences and associated features were first collected from the CPPsite 2.0 database [2]. We obtained the first dataset, composed of 1,855 entries corresponding to each entry to a CPP and their features in the dataset. The information retrieved from this database included the CPP identifier, its name, and corresponding sequence, along with information such as PubMed IDs, cell lines used in the study, and cargo coupled to the CPP. All scientific articles referenced in CPPsite2.0 were manually curated to fill POSEIDON with CPPs quantitative uptake values and respective units. Uptake values were recorded when quantitative data were available in plots or when they were directly mentioned by the authors. In addition, the temperature, concentration, time for CPP incubation, and uptake evaluation methods from the referenced articles were manually annotated. Therefore, only peptides with quantitative information were retained in the dataset, reducing the number of curated entries to 906, which corresponds to 676 unique CPPs.

Subsequently, we conducted a thorough literature search to supplement the database with manually curated samples. This process involved extensive and careful examination of relevant publications to identify additional data points. To this end, another 228 CPP-related articles from PubMed were queried using the filters “((((CPP) AND (Cell Penetrating Peptide)) OR (Cell-penetrating Peptide)) AND (Cellular Uptake)) AND ((”2015/11/19 “[Date—Publication]:”2022/08/01 “[Date—Publication])))”)” were evaluated and quantitative experimental information was added when existent. The final database comprised 2,371 entries, of which 1,315 were unique CPPs and 1,056 were CPPs with different uptake conditions. The latter refers to unique peptides that have been repeated under different conditions, such as varying cargoes, cell lines, temperatures, or incubation times, to analyze the uptake capacity of a peptide under different conditions.

To develop a suitable ML approach, it was necessary to refine the dataset to ensure the uniformity of the target variable (Uptake) in units, values, and experimental determination approaches. The following steps were performed to obtain a benchmark dataset for ML training and testing:

- Rows lacking information on concentration or with unclear peptide sequences were excluded, resulting in 2,067 remaining samples.
- Only samples determined by fluorescence were retained, as other methods would yield different target variables, leaving 1,765 samples.
- Samples with relative uptake efficiencies were excluded because they could not be interpreted as actual experimental values for ML purposes, reducing the dataset to 1,563 samples.
- Samples with unusable peptide concentration information were removed, leaving a final set of 1,316 samples.
- Peptide sequences that contained an excessive number of anomalous amino acids or non-peptide sequences were manually curated and excluded, resulting in 1,274 samples.
- Outliers for Uptake were removed, resulting in a final dataset size of 1,263 peptides.
- Since the original dataset had the same CPPs appearing multiple times but with different uptake conditions, we included these repetitions in the ML dataset. This was done because varying uptake conditions are considered important factors for developing an ML predictor. As a result, the ML dataset, consisting of 1,263 peptides, contained CPPs that appeared multiple times under different uptake conditions, totaling 642 unique CPPs.

The POSEIDON original dataset and the ML predictor dataset are available at the following GitHub repository: https://github.com/MoreiraLAB/poseidon/tree/main/data. These datasets are stored under the names “CPP_dataset.csv” and “CPP_ML.csv”, respectively.

### Feature extraction

To prepare the dataset for ML, the POSEIDON pipeline incorporates various features that aim to characterize peptides, cell lines, and experimental conditions.

The features can be further classified into three subcategories.

- Whole-peptide features were obtained using the Peptides R package [25].
- In-house position one-hot encoding features based on the size of the longest peptide. One-hot encoding is a reliable and interpretable method for representing categorical data such as amino acids in peptides [26, 27]. It is compatible with traditional ML algorithms, is robust to data variations, and minimizes information loss.
- Annotation-based features, in which the sequence anomaly type and location were substituted with the closest amino acids (Additional file 1: Table S1).

Cell line features (736 in total) were obtained from the Genomics of Drug Sensitivity in Cancer (GDSC) [28] database and matched with the cell lines of the POSEIDON dataset. They were then tagged as a true match depending on whether they were present on the GDSC. The POSEIDON dataset contained 43 available cell lines from the GDSC (Additional file 1: Table S2).

Finally, the experimental conditions were characterized using several variables (71 in total), including concentration (μM), categorical temperature (°C), incubation time availability and duration (in minutes), and curated cargo to avoid repetition (Additional file 1: Table S3). Prior to dimensionality reduction, this added up to 2,908 features (Table 1).

**Table 1:** POSEIDON features for the ML summary table

Total	Sample object	Amount	Description
2.908	Peptides	31	Whole-peptide features
		2.000	Peptide-position one-hot encoding. Considering the maximum size of 100 amino acids (longest peptide registered in the dataset), one-hot encoding was used for each of the positions of the 20 amino acids
		70	After inspecting the peptide sequences with anomalous amino acid substitutions, we annotated the position of the substitution (maximum of 24 as this was the size of the longest peptide with anomalous substitutions). Allows 56 possible substitutions along with those registered in the dataset
	Cell Lines	735	According to the GDSC, cell line gene mutation data includes 42 available cell lines
		1	According to the GDSC, cell line gene mutation data includes 42 available cell lines A categorical variable to indicate whether the cell line present in POSEIDON is exactly that of GDSC or a similar cell line present in the same tissue
	Experimental	1	Concentration (μM) of the peptide sequence
		5	Categorical temperature (°C). Although it is possible to use a numerical variable, there are only five available temperatures with biological relevance. For example, 37 °C is the regular human body temperature and 25 °C is a common room environment. For these reasons, and because in some cases, there is no temperature information available, the temperature was categorically encoded
		2	Incubation time and duration (min)
		63	Annotated cargo was manually curated in several steps of the dataset. Initially, only cargoes annotated in the original research papers were considered. Additionally, while processing the dataset, position-independent additions were considered as cargoes

### Data pre-processing and statistics treatment

Data cleaning, visualization, selection, and preprocessing of the raw dataset were performed using the programming language R (version 4.1.0) [29]. Peptides with unknown uptake values were excluded from the final dataset, as the methodologies used in these studies did not quantitatively measure peptide internalization. The resulting dataset consisted of 2,371 peptides with quantitative values, varying units, and uptake-evaluation techniques.

Subsequently, statistical analysis of the data was performed using RStudio (version 1.4.1717) [30]. The *tidyverse* package (version 1.3.1), which includes *dplyr* for data manipulation and *ggplot2* for data visualization [31], was used for the data analysis.

To construct the processed dataset, Python programming language (version 3.10.8) was used in combination with *NumPy* (version 1.24.1) and *Pandas* (version 1.5.2.), and *scikit-learn* (version 1.2.0). The usable samples were extracted and accessed on GitHub (https://github.com/MoreiraLAB/poseidon). The dataset underwent several uniformization steps such as incubation time uniformization, temperature encoding, valid peptide sequence generation, and curation of the target variable (peptide uptake) in log10 form, as it provides a more comprehensible scale.

Feature extraction was performed as described, resulting in 1,330 usable features after removing features with null variance, which can be fully explained and linked to real information, as depicted on the website. A random 70–30 data split was performed, and data normalization was applied based on the average and standard deviation of the training set, which was then applied to both the training and test sets. The decision to retain dimensionality without reduction was bolstered by several factors: the sample size of the dataset, the relevance of domain-specific features, the robust performance of the model on an independent test set encompassing 30% of the total data, the need for transparency to facilitate interpretability, and the model's evident ability to withstand overfitting despite its high dimensionality. Notably, this high dimensionality was driven by the inclusion of relevant one-hot encoding features that accounted for 98% of the feature space.

### Machine learning models deployment and optimization

After constructing the training and test sets as described, a battery of ML models from scikit-learn (1.2.0) [32] was implemented upon hyperparameter optimization (Table 3). In particular, xgboost (1.7.3) [33] and TensorFlow (2.11.0) [34] were optimized using ray[tune] (2.2.0) [35] as a tool (parameter range in Table 2). The tested models were a Support Vector Machine (SVM), Stochastic Gradient Descent (SGD), k-Nearest Neighbors (kNN), Decision Tree (DT), Random Forest (RF), Extreme Randomized Trees (ERT), eXtreme Gradient Boosting (XGB), Deep Neural Network (DNN), and forked Neural Network (fNN). While most of these models are standard imports from their respective packages, the fNN was designed for these purposes, comprising a neural network with different points of entry for each feature block type. All models were parameterized using the training set and an independent testing set. In this study, we evaluated the performance of our regression ML models using several metrics, including Root Mean Squared Error (RMSE), Mean Squared Error (MSE), Mean Absolute Error (MAE), Pearson correlation, Spearman correlation, and coefficient of determination (r^2^)-

**Table 2:** Hyper parameter optimization parameters for all the tested models

Model	Parameters	Package
Support vector machine	**Kernel:** [“linear”, “poly”,”rbf”,”sigmoid”]; **C:** [0.5, 1.0, 1.5]; **Gamma:** [“scale”, “auto”]	scikit-learn
Stochastic gradient descent	**Loss:** “squared_error”; **Penalty:** [“l2″, “l1″,”elasticnet”]; **Alpha:** [0.00001, 0.0001, 0.001]; **Learning rate:** [“invscaling”, “optimal”, constant”,”adaptive”]	
k-nearest neighbors	**N Neighbors:** [2, 3, 5, 7]; **P:** [1, 2]; **Algorithm:** [“auto”, “ball_tree”, “kd_tree”, “brute”]	
decision tree	**Splitter:** [“best”, “random”]; **Criterion:** ["squared_error", "friedman_mse", "absolute_error"]; **Maximum depth:** [None, 3, 5, 10, 50, 100]; **Minimum samples split:** [2, 3, 5, 7, 10]; **Minimum samples leaf**: [2, 3, 5, 7, 10]; **Minimum weight fraction leaf:** [0.0, 0.25, 0.50]; **Maximum features:** ["auto", "sqrt", "log2", None]	
Random forest	**Number of estimators:** [10, 50, 100, 250]; **Criterion:** ["squared_error", "friedman_mse", "absolute_error"]; **Maximum depth:** [3, 5, 10, 50, 100]; **Minimum samples split:** [2, 3, 5, 7, 10]; **Minimum samples leaf**: [2, 3, 5, 7, 10] **Minimum weight fraction leaf:** [0.0, 0.25, 0.50]	
Extreme randomized trees	**Number of estimators:** [10, 50, 100, 250]; **Criterion:** ["squared_error", "friedman_mse", "absolute_error"]; **Maximum depth:** [None, 3, 5, 10, 50, 100]; **Minimum samples split:** [2, 3, 5, 7, 10]; **Minimum samples leaf**: [2, 3, 5, 7, 10]; **Minimum weight fraction leaf:** [0.0, 0.25, 0.50]	
Extreme gradient boosting	**Number of estimators:** [10, 50, 100, 250]; **Maximum depth:** [None, 3, 5, 10, 50, 100]; **Maximum leaves:** [None, 1, 3, 5, 10, 25]; **Learning rate:** [None, 0.15, 0.3, 0.46, 0.60, 0.76, 0.90]; **Booster:** [None, "gbtree", "gblinear", "dart"]; **Alpha:** [0, 1, 3, 5]; **Lambda:** [1, 3, 5]; **Gamma:** [0, 1, 3, 5]	xgboost
Deep neural network	**Depth:** tune.qrandint(1, 10); **Layer size:** tune.qrandint(100, 1500, 100); **Use dropout:** tune.grid_search([True, False]); **Dropout rate:** tune.quniform(0.1, 0.9, q = 0.1); **Epochs:** tune.qrandint(100, 1000, 10); **Learning rate:** tune.quniform(0.00001, 0.001, q = 0.00001)	tensorflow
Forked neural network	**Depth:** tune.qrandint(1, 10); **Dropout:** tune.quniform(0.1, 0.9, q = 0.1); **Use dropout:** tune.grid_search([True, False]); **Learning Rate:** tune.quniform(0.00001, 0.001, q = 0.00001); **Experimental layer size:** tune.qrandint(5, 50); **Cargo layer size:** tune.randint(25, 250); **Sequence anomalies layer size:** tune.qrandint(5, 200); **Whole-peptide features layer size:** tune.qrandint(10, 300); **Sequence encoding layer size:** tune.qrandint(100, 1000); **Genomics layer size:** tune.qrandint(100, 750); **Anomalous position layer size:** tune.qrandint(5, 50); **Epochs:** tune.qrandint(100, 1000, 10)	

### POSEIDON front-end implementation

A web server free available to the scientific community can be found at https://moreiralab.com/resources/poseidon/. The webserver was constructed using the Nginx webserver with a Linux operating system. To develop the web interface, Flask [36] was used as the back end and HTML, CSS, and JavaScript were applied as the front end in conjunction with *Plotly* [37] for dynamic plot visualization.

Upon navigating to the POSEIDON platform, users are greeted with an intuitive interface designed to facilitate the submission of peptide sequences for prediction. Detailed instructions are provided on the homepage to guide users through the input process. This involves the following steps:

- Users input peptide sequence(s) into a designated text field within the interface.
- After entering the sequence, users can customize properties, such as peptide concentration, incubation time, temperature, and cell line type.
- Users are required to provide a valid email address to which the prediction results will be sent.
- To initiate the prediction process, users must click the 'Submit' button.

After submission, the POSEIDON prediction is swiftly computed, and the results are delivered on a separate page. Users are notified via email when a run succeeds.

Data and associated code underpinning the analyses presented herein are accessible via the repository at https://github.com/MoreiraLAB/poseidon.

## Results

### Database description

The POSEIDON database is a unique collection of recent information on CPPs, including quantitative cellular uptake values that have been experimentally obtained for each peptide. In addition to including all peptides in the CPPsite 2.0 database for which experimental quantitative cellular uptake data are available, POSEIDON has been highly enriched with up-to-date mining of the available literature.

A dataset of 2,371 entries was obtained through several steps of data acquisition and preprocessing, providing information about uptake evaluation methods, uptake conditions (such as temperature, cell line, and time of CPP incubation), uptake values, uptake units, cargoes, and peptide sequence. Both the CPPsite 2.0, and POSEIDON databases share information on peptide sequences, characteristics, modifications, validation methods, and cargo types. However, POSEIDON stands out because it offers quantitative uptake values for CPPs, whereas CPPsite 2.0 provides qualitative data.

**Fig. 2:**
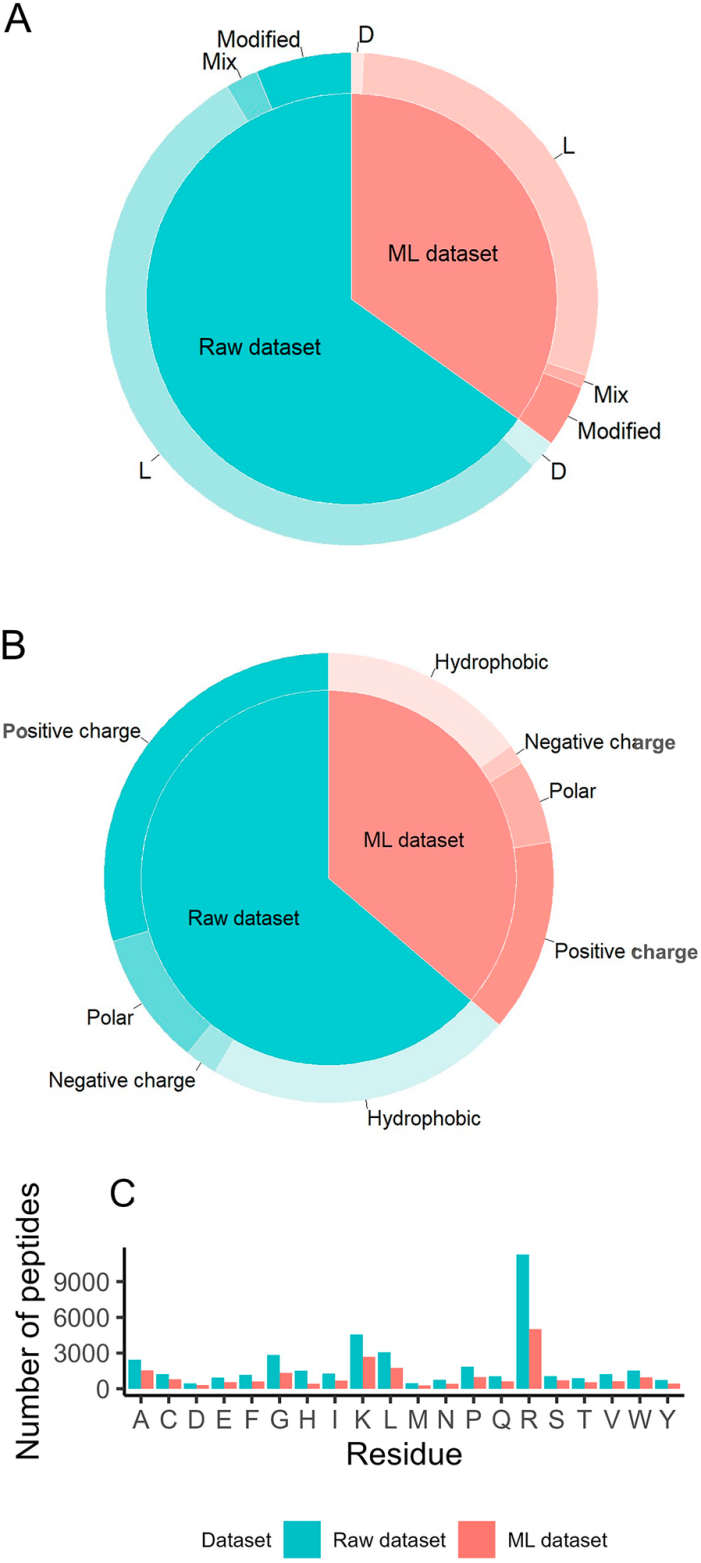
Representation of peptide composition in the POSEIDON database, raw data in blue, and benchmark data in red based on **A** chirality/modifications of CPP, **B** the type of amino acid, and **C** quantification of the amino acid composition of CPPs. The data pertain to peptides without non-natural amino acids

POSEIDON covers all types of CPPs, including L-amino acids, D-amino acids, L- and D-amino acids, and non-natural amino acids (Fig. 2A). The composition of CPPs revealed that certain types of residues, such as arginine, lysine, and leucine, were more prominent in CPPs than in methionine, aspartate, tyrosine, and asparagine residues, which were not enriched in CPPs (Fig. 2C). The positively charged residues like arginine and lysine in POSEIDON interact with negatively charged cell membrane components, increasing cellular uptake, as shown in Fig. 2B. The amphiphilic nature of CPPs, owing to their cationic and hydrophobic residues, enhances their interactions with the cell membrane and improves cell penetration [38] or cargo interaction [39].

**Fig. 3:**
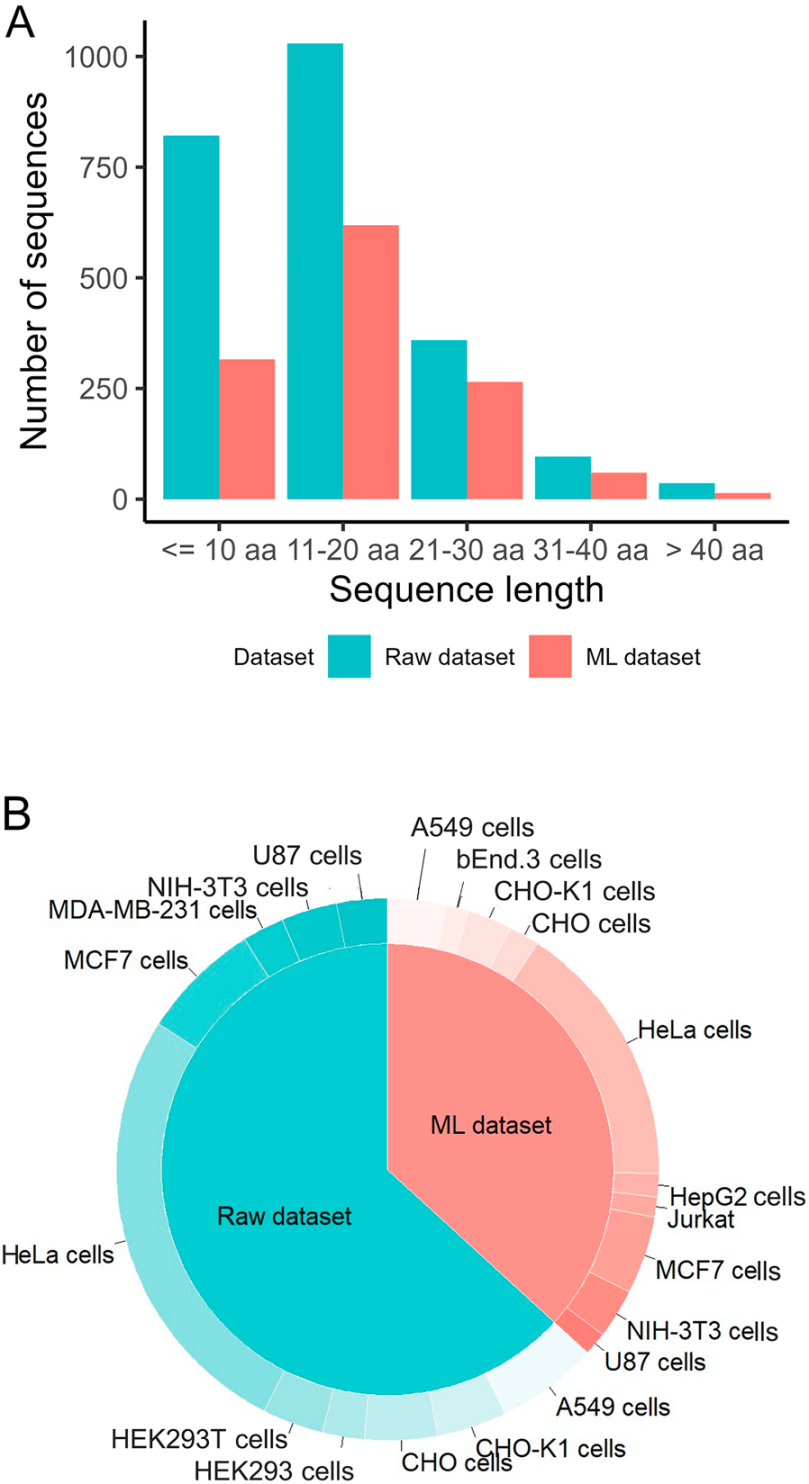
CPP features in both datasets (raw data in blue and benchmark data in red). **A** Length of peptide sequences in the database. **B** The 10 most used cell lines according to the dataset

This database provides peptide sequences that facilitate the retrieval of physicochemical properties that can be directly calculated from their primary sequences. Our dataset contained a significant number of peptides with lengths less than 10 amino acids (n = 821) and between 11 and 20 amino acids (n = 1,029), as shown in Fig. 3A. Most CPPs exhibit molecular weights ranging from 1 to 1.5 kDa. Both charge distribution and peptide length properties enable CPPs to interact with various cell-surface molecules, significantly influencing the selection of an entry pathway [40]. Among several influencing factors, such as the physicochemical properties of the peptide and its cargo, the internalization routes of CPPs are primarily directed towards two major pathways: endocytosis (an active or energy-dependent process) and membrane translocation (a direct or passive energy-independent process) [41]. Therefore, we analyzed the distribution of the cell lines, as they play a key role in peptide cell penetration. POSEIDON showed that more than 100 cell lines are associated with CPPs internalization. As shown in Fig. 3B, most CPPs were tested in HeLa cells (n = 597), followed by MCF7 (n = 162), A549 (n = 137), CHO (n = 97), CHO-K1 (n = 94), and HEK293T cells (n = 82). The diversity of cell lines ensures that CPP/cell line combinations can be analyzed using this database.

**Fig. 4:**
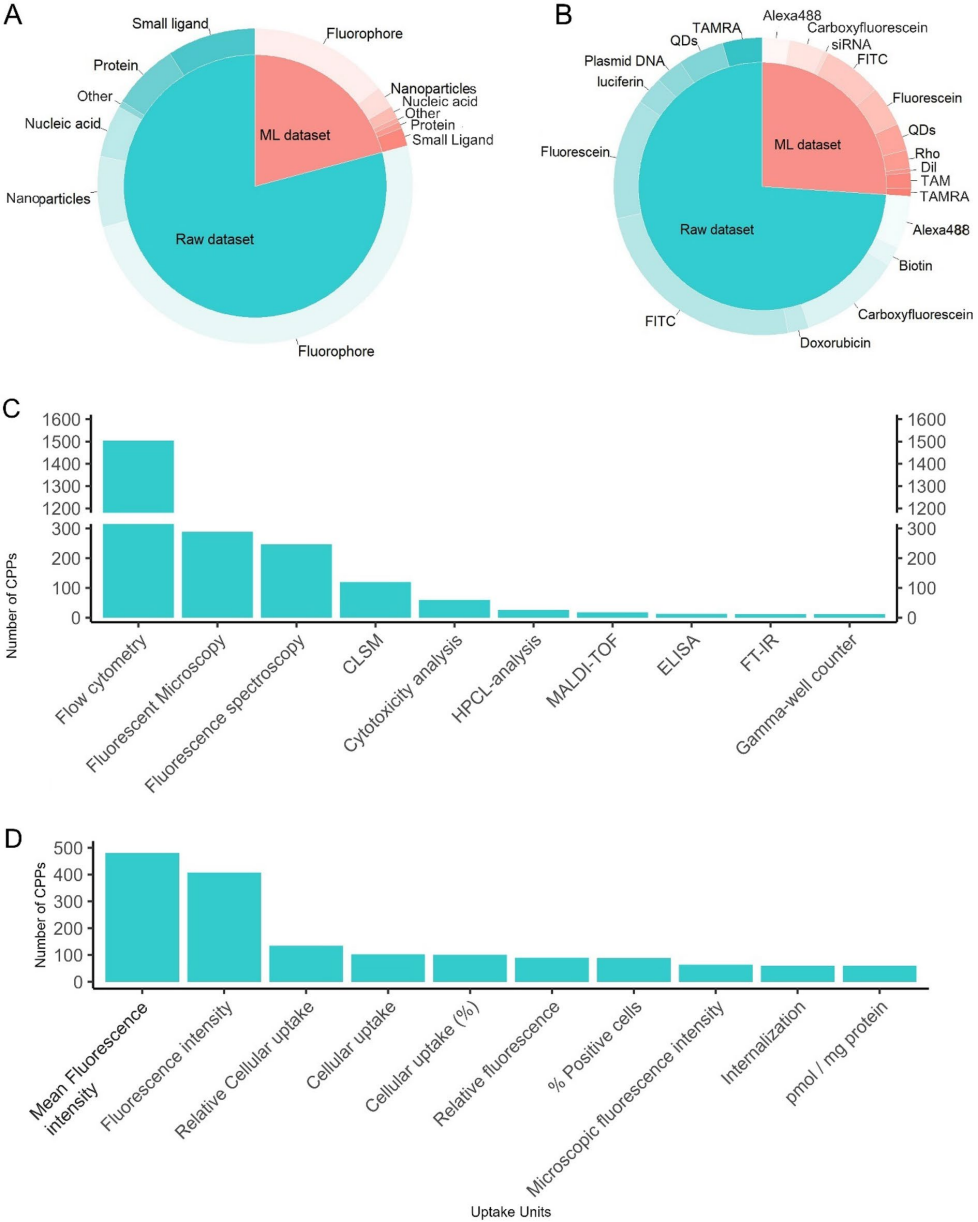
Distribution of CPPs in POSEIDON according to **A** cargo, **B** cargo type, **C** uptake evaluation methods, and **D** uptake units. **A** and **B** represent both datasets: raw data in blue and benchmark data in red

Scientific studies have shown that there are various roles associated with CPPs, ranging from fluorophores to nucleic acids. Thus, cargoes associated with each peptide are available in POSEIDON. As expected, our dataset demonstrated that fluorescein isothiocyanate (FITC), fluorescein, and carboxyfluorescein were the cargoes most strongly associated with CPPs (Fig. 4A). As shown in Fig. 4B, most CPPs in the dataset were associated with fluorophores (n = 4,368), followed by small ligands (n = 795), nanoparticles (n = 633), proteins (n = 600), and nucleic acids (n = 471).

Flow cytometry was the most commonly employed method for uptake evaluation in this dataset, accounting for 1,349 entries, whereas fluorescence microscopy, fluorescence spectroscopy, and Fluorescence-Activated Cell Sorting (FACS) were employed for 289, 247, and 155 entries, respectively (Fig. 4C). However, as shown in Fig. 4D, there was a high degree of variability in the uptake units, and several studies used slight differences in identical uptake unit designations.

After standardizing identical units to a unique designation, the mean fluorescence intensity was the most frequently employed unit in this dataset, with 481 entries. The different units presented in Fig. 4C highlight the lack of standardization in CPP uptake evaluations conducted in previous studies, which hinders the comparison and analysis of the CPP uptake data. Although there are currently no standardized methods for CPP uptake evaluation, flow cytometry has been employed significantly more frequently than the other methods. This suggests that it is possible to establish a general method using specific easily attainable controls, allowing a large amount of quantitative data to be acquired and compared more adequately and easily. This database also provides information on the temperature and time of CPP incubation. Due to the nature of CPPs and their internalization mechanisms, changes in certain conditions, such as temperature, can significantly impact the uptake of CPPs by cells, often due to alterations in the underlying mechanism [42–44]. Thus, these data are highly valuable for the development of new approaches.

### Processed database description

The POSEIDON database uptake-prediction methods developed in this study rely exclusively on fluorescence measurements. This approach was selected because other methods can produce inconsistent results, leading to discrepancies in the derived uptake units. Therefore, to establish a reliable benchmark dataset, we selected CPPs that were evaluated using fluorescence methods,

resulting in a dataset of 1274 entries. After removing outliers, the final dataset contained 1263 entries.

As shown in red in the figures, most amino acids are L-amino acids (Fig. 2A) and were essentially hydrophobic and polar charged (Fig. 2B). Similar to the raw dataset, arginine, lysine, and leucine were present in large numbers in the CPP sequences, in contrast to methionine, aspartate, asparagine, and tyrosine residues, which were not prominent in CPPs (Fig. 2C).

The benchmark dataset included CPP sequences of various sizes, with sequences consisting of 11–20 residues being the most common (n = 619), followed by sequences with fewer than 10 residues (n = 316), and sequences consisting of 21–30 residues (n = 265) (Fig. 3A, red). In terms of cell lines, HeLa cells were the most frequently used, as in the raw dataset. However, the benchmark dataset showed the emergence of HepG2, Jurkat, and bEnd.3 cell lines as among the most frequently used cell lines for CPPs. Regarding cargo, the benchmark dataset showed a slightly different trend than the raw dataset, with Dil, rhodamine (Rho), small interfering RNA (siRNA), and TAM being highly associated with CPPs. Fluorophores were the most common cargo (n = 1,249), followed by nanoparticles (n = 198), small ligands (n = 165), nucleic acids (n = 110), and proteins (n = 56) (Fig. 4B, red).

Additional interesting information emerges when conducting a correlation analysis between the features and the processed target variable. Among the 30 features that exhibited the highest correlation with the target variable (Additional file 1: Table S4), 50% with the highest Pearson correlation were position-encoding features. One-third of the most correlated features are genomic features. Only two features from the entire sequence were present in the top 30, whereas cargo had 3. Although experimental features such as concentration and temperature were not included in the top 30, it is apparent that they are among the top 100 on the additional figures on the website.

### Performance of the different predictors

After implementing the hyperparameter optimization pipeline (Table 3**)**, the best-performing models were XGB and DNN, as indicated by their evaluation metrics on the independent test set that did not participate in either training or hyper-parameter optimization (Table 4). Specifically, both models achieved high r^2^ scores, exceeding 0.76, whereas the other methods barely surpassed the 0.70 threshold. Furthermore, they exhibited high correlation metrics, with Pearson correlations above 0.87 and Spearman correlations above 0.88. Consequently, the final prediction pipeline of POSEIDON displays predictions generated by both DNN and XGB models.

**Table 3:** Optimal parameters for optimized ML models

Model	Parameters	Package
Support vector machine	**Kernel:**”rbf”; **C:** 1.5; **Gamma:** “scale”	scikit-learn
Stochastic gradient descent	**Loss:** “squared_error”; **Penalty:** “l2″; **Alpha:** 0.00001; **Learning rate:**”adaptive”	
k-nearest neighbors	**N Neighbors:** 2; **P:** 2; **Algorithm:** “brute”	
Decision tree	**Splitter:** “best”; **Criterion:** "friedman_mse"; **Maximum depth:** 10; **Minimum samples split:** 3; **Minimum samples leaf**: 7; **Minimum weight fraction leaf:** 0.0; **Maximum features:** "auto"	
Random forest	**Number of estimators:** 50; **Criterion:** "squared_error"; **Maximum depth:** 50; **Minimum samples split:** 3; **Minimum samples leaf**: 3; **Minimum weight fraction leaf:** 0.0	
Extreme randomized trees	**Number of estimators:** 10; **Criterion:** "friedman_mse"; **Maximum depth:** 100; **Minimum samples split:** 10; **Minimum samples leaf**: 7; **Minimum weight fraction leaf:** 0.0	
Extreme gradient boosting	**Number of estimators:** 50; **Maximum depth:** 10; **Maximum leaves:** 10; **Learning rate:** None; **Booster:** "dart"; **Alpha:** 1; **Lambda:** 3; **Gamma:** 0	xgboost
Deep neural network	**Depth:** 1; **Layer size:** 500; **Use dropout:** True; **Dropout rate:** 0.3; **Epochs:** 230; **Learning rate:** 0.0005	tensorflow
Forked neural network	**Depth:** 7; **Dropout:** 0.9; **Use dropout:** False; **Learning Rate:** 0.0001; **Experimental layer size:** 39; **Cargo layer size:** 239; **Sequence anomalies layer size:** 79; **Whole-peptide features layer size:** 155; **Sequence encoding layer size:** 850; **Genomics layer size:** 687; **Anomalous position layer size:** 45; **Epochs:** 170

**Table 4:** Results for the best performance of each optimized ML model

Model	Subset	RMSE	MSE	MAE	Pearson	Spearman	r^2^
Support vector machine	Train	0.550	0.303	0.318	0.918	0.943	0.817
	Test	0.717	0.514	0.485	0.856	0.887	0.706
Stochastic gradient descent	Train	–	–	–	0.002	0.002	–
	Test	–	–	–	0.002	0.002	–
k-nearest neighbors	Train	0.419	0.175	0.263	0.946	0.942	0.894
	Test	0.746	0.557	0.572	0.828	0.823	0.681
Decision tree	Train	0.690	0.476	0.488	0.844	0.824	0.712
	Test	0.951	0.904	0.669	0.704	0.707	0.483
Random forest	Train	0.397	0.158	0.259	0.958	0.961	0.905
	Test	0.700	0.490	0.452	0.856	0.869	0.720
Extreme randomized trees	Train	0.527	0.277	0.349	0.915	0.924	0.832
	Test	0.765	0.585	0.522	0.819	0.830	0.665
Extreme gradient boosting	Train	0.177	0.031	0.098	0.991	0.989	0.981
	Test	0.643	0.413	0.394	0.874	0.881	0.764
Deep neural network	Train	0.259	0.067	0.153	0.980	0.979	0.959
	Test	0.640	0.410	0.402	0.876	0.880	0.765
Forked neural network	Train	0.358	0.128	0.199	0.961	0.960	0.923
	Test	0.740	0.547	0.447	0.839	0.857	0.687

## Discussion

CPPs have great potential in therapy and diagnosis; however, identifying new and efficient CPPs can be costly and time-consuming. Consequently, computational biological studies have become increasingly important in this field, although they have mainly focused on the qualitative features of CPPs. POSEIDON addresses this gap by offering a novel up-to-date database that includes quantitative experimental uptake efficiency data and serves as a benchmark for the field. The POSEIDON database and prediction pipeline have provided several important insights into the rapidly evolving field of CPP research. First, it is evident that effective CPPs are characterized by an abundance of positively charged amino acids, which is biochemically logical because it allows peptides to leverage the electrostatic differences inside and outside the cell, thereby augmenting cellular internalization. Indeed, the internalization mechanism of CPPs remains a subject of ongoing debate, with CPP concentration, charge, and amphipathicity emerging as crucial factors. The intricate processes governing CPP internalization involve a combination of endocytic and direct translocation mechanisms [41]. The positive charge, particularly from arginine residues, significantly influenced CPP uptake, with arginine being more favorable for delivery and CPP activity than lysine. Amphipathicity peptides can directly penetrate the cell membrane at low concentrations, whereas non-amphipathic CPPs rely on endocytosis [6]. Regarding CPP concentration, endocytosis is typically the predominant mechanism under physiological conditions and at low peptide concentrations. In contrast, at higher peptide concentrations, direct translocation across the plasma membrane becomes more prevalent [41]. Further investigation of the specific mechanisms employed by CPPs with different physicochemical properties and concentrations will provide valuable insights into the complex dynamics governing cellular uptake.

Second, fluorophores are significant molecular interventions for CPP activity, as their presence is methodologically required, and they are highly correlated with the uptake variable, implying that they may intervene in molecular interactions. Moreover, the presence of cargo can modify the CPP uptake pathway, as demonstrated by the observed impact of cargo size and binding methodology on the CPP translocation mechanism [41, 45].

Third, genomics descriptors play a crucial role in this process, which was not adequately addressed before POSEIDON. Notably, mutation of the NRAS gene, which is linked to cell division in cancer, was found to be the variable most correlated with CPP uptake, followed closely by mutation of IDH1, which is associated with the expression of isocitrate dehydrogenase 1, a key player in the Krebs Cycle. Exploring the biological relationship between these genes (and several others high in ranking) and CPPs might be a worthy endeavor.

Fourth, CPP penetration into cells is influenced by the cell line owing to differences in membrane composition, receptor expression, and intracellular mechanisms. These factors affect the effectiveness and penetration mechanism of CPPs. Understanding CPP behavior in specific cell lines is crucial for accurate results, as the findings may not apply universally, as studies on various cell lines reveal cell-dependent preferences for specific CPPs [41], which also supports targeted CPP application in various biological and therapeutic contexts.

The POSEIDON database is not only the largest but also a comprehensive, curated database with CPP information. The inclusion of an extensive range of experimental characteristics in our dataset underscores the complexity inherent in CPP behavior. The prediction method employed by POSEIDON is unique in that it effectively considers CPP uptake activity as a continuous variable, unlike previous efforts that only featured categorical predictions. Our approach also includes multiple previously unused sources of information, which will allow users to test sequence anomalies, select tissue-specific cell lines, choose up to two cargoes per peptide, and adjust experimental conditions, such as temperature, concentration, and incubation time. We ensured that the algorithm incorporated all relevant parameters, thereby enabling it to capture intricate and nonlinear relationships among the variables. This approach enhances the predictive capacity of the model, making it adept at handling multifaceted experimental conditions encountered in various studies.

Assessing the POSEIDON ML approach in comparison with other prediction methods poses a distinct challenge mainly because of the limited availability of similar approaches. Nonetheless, Dowaidar et al. represented an exception, as they spearheaded the creation of Fragment Quantitative Structure–Activity Relationship (FQSAR) models [46]. These models were specifically tailored to forecast the biological activity of CPPs in peptide-based transfection systems (PBTS), trained on only 11 data points, yet achieved r^2^ values ranging from 0.906 to 0.961 across various models. Nevertheless, POSEIDON stands out with very high correlation metrics and low errors, fully demonstrating its ability to predict CPP uptake under different conditions with exceptional performance.

## Conclusion

POSEIDON provides the first quantitative data on cellular uptake, methodology, units, and experimental conditions, making it an exceptional tool. The POSEIDON database, a recently launched, open-source, and comprehensive resource, focuses exclusively on curated CPPs with quantitative uptake values. Each CPP in the database is accompanied by physicochemical properties, cell line, cargo, sequence, uptake evaluation method, concentration, temperature, and incubation time. The POSEIDON predictor is also groundbreaking, as it was the first tool to predict CPP uptake based on quantitative uptake and genomic data. With its dynamic, free, and easy-to-use interface, users can easily submit a peptide sequence and obtain computational predictions of its uptake in various cell lines. Additionally, users can customize properties, such as peptide concentration, incubation time, temperature, and cell line type. The POSEIDON database is a unique resource for researchers to develop new methodologies and predictors for CPP sequence design, based on uptake values.

### Supplementary Information

**Additional file 1: Table S1.** Possible anomalies and their substituted amino acids after annotation. **Table S2.** Tissue name, available GDSC cell lines, corresponding POSEIDON cell lines (if the same cell line overlapped in both datasets, in bold). **Table S3.** Annotated cargo tables with unique names that appear in the POSEIDON dataset, possible literature names, and those that remain the same. **Table S4.** The top 30 features were highly correlated with the target variable, log10(uptake).

## Data Availability

All data can be found at www.moreiralab.com/resources/poseidon and https://github.com/MoreiraLAB/poseidon).

## References

[1] Xie J, Bi Y, Zhang H (2020). Cell-penetrating peptides in diagnosis and treatment of human diseases: from preclinical research to clinical application. Front Pharmacol.

[2] Agrawal P, Bhalla S, Usmani SS (2016). CPPsite 2.0: a repository of experimentally validated cell-penetrating peptides. Nucleic Acids Res.

[3] Kristensen M, Birch D, Mørck Nielsen H (2016). Applications and challenges for use of cell-penetrating peptides as delivery vectors for peptide and protein cargos. Int J Mol Sci.

[4] Xu J, Khan AR, Fu M (2019). Cell-penetrating peptide: a means of breaking through the physiological barriers of different tissues and organs. J Control Release.

[5] Habault J, Poyet J-L (2019). Recent advances in cell penetrating peptide-based anticancer therapies. Molecules.

[6] Madani F, Lindberg S, Langel U (2011). Mechanisms of cellular uptake of cell-penetrating peptides. J Biophys.

[7] Yang J, Luo Y, Shibu MA (2019). Cell-penetrating peptides: efficient vectors for vaccine delivery. Curr Drug Deliv.

[8] Porosk L, Põhako K, Arukuusk P, Langel Ü (2021). Cell-penetrating peptides predicted from CASC3, AKIP1, and AHRR proteins. Front Pharmacol.

[9] Derakhshankhah H, Jafari S (2018). Cell penetrating peptides: a concise review with emphasis on biomedical applications. Biomed Pharmacother.

[10] Milletti F (2012). Cell-penetrating peptides: classes, origin, and current landscape. Drug Discov Today.

[11] Koo J-H, Kim G-R, Nam K-H, Choi J-M (2022). Unleashing cell-penetrating peptide applications for immunotherapy. Trends Mol Med.

[12] Ugalde-Triviño L, Díaz-Guerra M (2021). PSD-95: an effective target for stroke therapy using neuroprotective peptides. Int J Mol Sci.

[13] Samec T, Boulos J, Gilmore S (2022). Peptide-based delivery of therapeutics in cancer treatment. Mater Today Bio.

[14] European Medicines Agency (2020) EU/3/20/2328 - orphan designation for treatment of Friedreich’s ataxia. https://www.ema.europa.eu/en/medicines/human/orphan-designations/eu-3-20-2328. Accessed 30 Aug 2023

[15] Gao S, Simon MJ, Hue CD (2011). An unusual cell penetrating peptide identified using a plasmid display-based functional selection platform. ACS Chem Biol.

[16] Lee J-H, Song HS, Park TH (2012). Screening of cell-penetrating peptides using mRNA display. Biotechnol J.

[17] Tang H, Su Z-D, Wei H-H (2016). Prediction of cell-penetrating peptides with feature selection techniques. Biochem Biophys Res Commun.

[18] Wei L, Xing P, Su R (2017). CPPred-RF: a sequence-based predictor for identifying cell-penetrating peptides and their uptake efficiency. J Proteome Res.

[19] Wei L, Tang J, Zou Q (2017). SkipCPP-Pred: an improved and promising sequence-based predictor for predicting cell-penetrating peptides. BMC Genomics.

[20] Kumar V, Agrawal P, Kumar R (2018). Prediction of cell-penetrating potential of modified peptides containing natural and chemically modified residues. Front Microbiol.

[21] Manavalan B, Subramaniyam S, Shin TH (2018). Machine-learning-based prediction of cell-penetrating peptides and their uptake efficiency with improved accuracy. J Proteome Res.

[22] Manavalan B, Patra MC (2022). MLCPP 2.0: an updated cell-penetrating peptides and their uptake efficiency predictor. J Mol Biol.

[23] Pandey P, Patel V, George NV, Mallajosyula SS (2018). KELM-CPPpred: Kernel extreme learning machine based prediction model for cell-penetrating peptides. J Proteome Res.

[24] Fu X, Cai L, Zeng X, Zou Q (2020). StackCPPred: a stacking and pairwise energy content-based prediction of cell-penetrating peptides and their uptake efficiency. Bioinformatics.

[25] Osorio D, Rondón-Villarreal P, Torres R (2015). Peptides: a package for data mining of antimicrobial peptides. R J.

[26] Wang D, Zeng S, Xu C (2017). MusiteDeep: a deep-learning framework for general and kinase-specific phosphorylation site prediction. Bioinformatics.

[27] Zhao J, Jiang H, Zou G (2022). CNNArginineMe: a CNN structure for training models for predicting arginine methylation sites based on the One-Hot encoding of peptide sequence. Front Genet.

[28] Yang W, Soares J, Greninger P (2013). Genomics of Drug Sensitivity in Cancer (GDSC): a resource for therapeutic biomarker discovery in cancer cells. Nucleic Acids Res.

[29] R Core Team (2021) R: A language and environment for statistical computing. R Foundation for Statistical Computing, Vienna, Austria. https://www.R-project.org/

[30] RStudio. http://www.rstudio.com/. Accessed 31 Jan 2022

[31] Wickham H, Averick M, Bryan J (2019). Welcome to the tidyverse. J Open Source Softw.

[32] Pedregosa F, Varoquaux G, Gramfort A, et al (2012) Scikit-learn: Machine Learning in Python. arXiv [cs.LG]

[33] Chen T, Guestrin C (2016) XGBoost: A Scalable Tree Boosting System. arXiv [cs.LG]

[34] Abadi M, Barham P, Chen J, et al (2016) TensorFlow: A system for large-scale machine learning. arXiv [cs.DC]

[35] Liaw R, Liang E, Nishihara R, et al (2018) Tune: A research platform for distributed model selection and training. arXiv [cs.LG]

[36] Rinberg M (2018) Flask web development: developing web applications with python

[37] Inc PT (2015). Collaborative data science.

[38] Silva S, Almeida A, Vale N (2019). Combination of cell-penetrating peptides with nanoparticles for therapeutic application: a review. Biomolecules.

[39] McClorey G, Banerjee S (2018). Cell-penetrating peptides to enhance delivery of oligonucleotide-based therapeutics. Biomedicines.

[40] Wang F, Wang Y, Zhang X (2014). Recent progress of cell-penetrating peptides as new carriers for intracellular cargo delivery. J Control Release.

[41] Ruseska I, Zimmer A (2020). Internalization mechanisms of cell-penetrating peptides. Beilstein J Nanotechnol.

[42] Dougherty PG, Sahni A, Pei D (2019). Understanding cell penetration of cyclic peptides. Chem Rev.

[43] Fretz MM, Penning NA, Al-Taei S (2007). Temperature-, concentration- and cholesterol-dependent translocation of L- and D-octa-arginine across the plasma and nuclear membrane of CD34+ leukaemia cells. Biochem J.

[44] Mueller J, Kretzschmar I, Volkmer R, Boisguerin P (2008). Comparison of cellular uptake using 22 CPPs in 4 different cell lines. Bioconjug Chem.

[45] Tünnemann G, Martin RM, Haupt S (2006). Cargo-dependent mode of uptake and bioavailability of TAT-containing proteins and peptides in living cells. FASEB J.

[46] Dowaidar M, Regberg J, Dobchev DA (2017). Refinement of a quantitative structure–activity relationship model for prediction of cell-penetrating peptide based transfection systems. Int J Pept Res Ther.

