# Supplementary material for: POSEIDON: Peptidic Objects SEquence-based Interaction with cellular DOmaiNs: a new database and predictor
Source: J Cheminform. 2024 Feb 16;16:18. doi: 10.1186/s13321-024-00810-7 (PMC10874016; doi:10.1186/s13321-024-00810-7)
Supplement: Supplementary file 1 — Additional file 1: Table S1. Possible anomalies and their substituted amino acids after annotation. Table S2. Tissue name, available GDSC cell lines, corresponding POSEIDON cell lines (if the same cell line overlapped in both datasets, in bold). Table S3. Annotated cargo tables with unique names that appear in the POSEIDON dataset, possible literature names, and those that remain the same. Table S4. The top 30 features were highly correlated with the target variable, log10(uptake). [file 13321_2024_810_MOESM1_ESM.docx]

# POSEIDON: Peptidic Objects SEquence-based Interaction with cellular DOmaiNs – A new database and predictor

António J. Preto^1,2^, Ana B. Caniceiro^1,3,4^, Francisco Duarte^1^, Hugo Fernandes^5,6,7^, Lino Ferreira^5,6^, Joana Mourão^5^ and Irina S. Moreira^3,5^*

1 Center for Neuroscience and Cell Biology, University of Coimbra, 3004-504 Coimbra, Portugal

2 PhD Programme in Experimental Biology and Biomedicine, Institute for Interdisciplinary Research (IIIUC), University of Coimbra, Casa Costa Alemão, 3030-789 Coimbra, Portugal

3 Department of Life Sciences, University of Coimbra, Calçada Martim de Freitas, 3000-456 Coimbra, Portugal

4 PhD Programme in Biosciences, Department of Life Sciences, University of Coimbra, Calçada Martim de Freitas, 3000-456 Coimbra, Portugal

5 CNC - Center for Neuroscience and Cell Biology & CIBB - Centre for Innovative Biomedicine and Biotechnology, University of Coimbra, Coimbra, Portugal

6 FMUC - Faculty of Medicine, University of Coimbra, Coimbra, Portugal

7 MIA – Multidisciplinary Institute of Ageing, University of Coimbra, Portugal.

Corresponding author: Irina S. Moreira, Biocant Park Parque Tecnológico de Cantanhede Núcleo 04, Lote 8, 3060-197 Cantanhede, Portugal; Tel.: (+351) 231 249 170; E-mail: irina.moreira@cnc.uc.pt.

**Supplementary data**

**Table S1.** Possible anomalies and their substituted amino acids after annotation.

| **In-Sequence** | **Anomaly substitution name** | **Substitute** |
| --- | --- | --- |
| 4Iph | 4-para-iodo-benzoyl | A |
| 4Iph_yl | 4-para-iodo-benzyl | A |
| Abu | alfa-amino-n-butyric acid | C |
| Ac | acetyl | G |
| Ac5c | 1-aminocyclopentane-1-carboxylic_acid | P |
| Ac5cdOM | (3S-4S)-1-amino-3-4-dimethoxycyclopentanecarboxylic_acid | P |
| Ace | acetamide | A |
| AChex | aminocyclohexane_carboxylic_acid | A |
| ACpen | aminocyclopentane_carboxylic_acid | A |
| Ado | dioxo_octanoic_acid | A |
| aeLP_linker | L_aminoethylprolyl_linker | P |
| aeDP_linker | D_aminoethylprolyl_linker | P |
| apLP_linker | L_aminopropylprolyl_linker | P |
| apDP_linker | D_aminopropylprolyl_linker | P |
| Ahx | aminohexanoic_acid | K |
| Aib | alfa-aminoisobutyric_acid | A |
| betaA | beta_alanine | A |
| Cbz | carbamate | A |
| cPEP | cRGDfC | RGDfC |
| Cys_linker | cysteine_linker | C |
| Fmoc | 9-fluorenylmethoxycarbonyl | W |
| Gly_linker | glycine_linker | G |
| Hbut | histidine_butyl | h |
| Hcy | homocysteine | C |
| Het | histidine_ethyl | h |
| Hiso | histidine_isopropyl | h |
| Hme | histidine_methyl | h |
| hydroP | hydroxy_proline | P |
| LNap | L-2-naphthylalanine | A |
| Lys_aet | lys_aet | k |
| Lys_get | lys_get | k |
| MeLeu | (S)-alpha-methylleucine | L |
| miniPEG_linker | miniature_polyethylene_glycol_linker | A |
| NIA | nitroimidazol_acetic_acid | A |
| Nle | norleucine | L |
| PABC_linker | mc_vc_pabc_linker | A |
| PEG2 | 2-unit_ethylene_glycol_spacer | A |
| R8 | r8_r_octenylalanine | A |
| S8 | s8_s_octenylalanine | A |
| S5 | s5_s_pentenylalanine | A |

**Table S2.** Tissue name, available GDSC cell lines, corresponding POSEIDON cell lines (if the same cell line overlapped in both datasets, in bold).

| **Tissue name** | **GDSC cell lines** | **POSEIDON cell lines** |
| --- | --- | --- |
| Acute Myeloid Leukaemia | KG-1 | **KG-1** |
| Bladder | CAL-29, T24 | ECV-304 |
| Breast | MCF7, MDA-MB-231 | **MCF7, MDA-MB-231,** MCF-7/Adr |
| Cervix | Ca-Ski, HeLa | **HeLa, Ca-Ski,** HEp-2, eGFP HeLa 654 |
| Chronic Myeloid Leukaemia | K-562 | **K-562** |
| Endometrium | AN3-CA | EJ |
| Fibrosarcoma | HT-1080 | NIH-3T3 |
| Glioma | 42-MG-BA, LN-18, U251, U-87-MG | **LN-18, U251, U-87-MG,** 9L, 9L-R, U87, D54, U373MG, F98 |
| Kidney | 769-P | HEK-293T, HEK-293, PK15 |
| Large Intestine | C2BBe1, HCT-116, HT-29 | **HCT-116, HT-29,** Caco-2, Vero, Cos-7, Lovo |
| Leukaemia | KY821 | MonoMac6, RAW264.7, RBL-2H3 |
| Liver | C3A | **C3A,** HepG2 |
| Lung NSCLC | A549 | **A549** |
| Lung NSCLC not specified | A427 | MRC-9 |
| Lymphoblastic leukaemia | CCRF-CEM | **CCRF-CEM** |
| Lymphoid neoplasm | YT | Hs 697.Sp |
| Melanoma | 451Lu, A101D, A375, SK-MEL-1 | **A375,** A375-R, HaCaT, MDA-MB-435S, MM96L, 3T3-L1 |
| Mesothelioma | MSTO-211H | MSTO, MSO-R |
| Myeloma | ARH-77 | Bone marrow dendritic |
| Neuroblastoma | BE2-M17 | BCECs, bEnd.3, HBMEC |
| Osteosarcoma | Saos-2, U-2-OS | **Saos-2, U-2-OS** |
| Ovary | A2780, Caov-3, SK-OV-3 | **A2780, SK-OV-3,** CHO-K1, pgsA-745, CHO, Ovcar-3, C3H10T1/2, 3T3, CHO pgsB-618, NCI/ADR, NIH-3T3 |
| Prostate | 22RV1, DU-145, PC-3 | **DU-145, PC-3**, LNCaP |
| Skin other | A388 | NHDF, HDF |
| T-cell leukaemia | Jurkat | **Jurkat,** Jurkat E6.1 leukaemia, Jurkat E6 |
| Other urogenital system | JAR | SV-HUC |

**Table S3.** Annotated cargo tables with unique names that appear in the POSEIDON dataset, possible literature names, and those that remain the same.

| **Unique Name** | **Possible literature names** |
| --- | --- |
| Alexa Dye | A488, a489, a490, a491, a492, a493, alexa, alexafluor555 |
| ATTO Dye | at520, at521 |
| Adivin | avidin, sa, streptavidin |
| Cyanine Dye | Cy3-labeled Crotamine, Cy5 |
| DNR | daunorubicin |
| DOX | Doxorubicin, SS-DOX |
| dsRBD | Hph1Hph1dsRBD |
| ELP-DOXO | ELP-GFLG-NCDox |
| FAM Dye | 5-fam, carboxyfluorescein, cf, fam |
| FITC Dye | FITC |
| Flu | fluorescein |
| GFP Dye | eGFP, GFP |
| IR Dye | IR800 |
| Liposomes | Liposomes, TSL |
| MS | Microspheres |
| pCAP | pCAP, pCP |
| QDots | QD, quantum dots |
| Rhod | Rho, Rhod, rhodamine |
| siRNA | GAPDH siRNA, siRNA |
| SNAP_enzyme | SNAP |
| Streptolysin-O | SLO |
| TAMRA Dye | 6-carboxy-tetramethylrhodamine, tmr, tam, tamra |
| TAZ | PDZ, TAZ |
| TR Dye | Texas Red |
| Transferrin | Tf |
| **Unchanged cargoes** | |
| AF568-SSO, Albumin, BH3, CD63, CLB, CmLN, CROP, CT-B, Dextran, Dil, DKP-RGD, EBD-S11, ELPBC, Folic acid, galactose, galactosidase, glucose, glucuronidase, GpYEEI, H, HKII, HPMA, Insulin-PEG-LMWP, KLA, mannose, mCherry Dye, MTS, MTSL, MTSLR, Natamycin, OH, Oleyl, oligonucleotides, PA, Phage, plasmid, PMD, trehalose, NDB | |

**Table S4.** **T**op 30 features highly correlated with the target variable log10(uptake).

| **Feature** | **Correlation** | **Feature Type** |
| --- | --- | --- |
| Gene NRAS mutation | 0.308 | Genomics |
| FITC dye | 0.281 | Cargo |
| Gene IDH1 mutation | 0.231 | Genomics |
| Lysine in position 9 | 0.208 | Position |
| Cysteine in position 7 | 0.195 | Position |
| Arginine in position 17 | 0.184 | Position |
| Gene cnaPANCAN343 | 0.177 | Genomics |
| Histidine in position 5 | 0.174 | Position |
| Gene cnaPANCAN342 | 0.174 | Genomics |
| Gene cnaPANCAN341 | 0.174 | Genomics |
| Frequency of aromatic amino acids | 0.174 | Whole-sequence |
| Asparagine in position 15 | 0.174 | Position |
| Gene cnaPANCAN340 | 0.165 | Genomics |
| PMO | 0.162 | Cargo |
| Lysine in position 10 | 0.161 | Position |
| Arginine in position 4 | 0.159 | Position |
| Gene BRWD1 mutation | 0.157 | Genomics |
| Tryptophan in position 6 | 0.157 | Position |
| Cysteine in position 12 | 0.152 | Position |
| Phenylalanine in position 11 | 0.152 | Position |
| Leucine in position 20 | 0.152 | Position |
| Histidine in position 12 | 0.152 | Position |
| Lysine in position 6 | 0.148 | Position |
| Gene cnaPANCAN336 | 0.147 | Genomics |
| HKII | 0.147 | Cargo |
| Number of aromatic amino acids | 0.141 | Whole-sequence |
| Phenylalanine in position 10 | 0.140 | Position |
| Gene cnaPANCAN382 | 0.140 | Genomics |
| Gene cnaPANCAN21 | 0.140 | Genomics |
| Tryptophan in position 3 | 0.139 | Position |
